# Generational Expression of Muir-Torre Syndrome in a Canadian Family

**DOI:** 10.1155/2016/1712527

**Published:** 2016-10-16

**Authors:** Kaitlin Alexandra Vanderbeck, R. Gary Sibbald, Nirosha Murugan

**Affiliations:** ^1^Department of Medicine, Northern Ontario School of Medicine, Laurentian University, 935 Ramsey Lake Road, Sudbury, ON, Canada P3E2C6; ^2^Department of Medicine, University of Toronto, Women's College Hospital, 76 Grenville St., Toronto, ON, Canada M5S 1B2; ^3^Department of Biomolecular Sciences, Laurentian University, 935 Ramsey Lake Road, Sudbury, ON, Canada

## Abstract

Muir-Torre syndrome (MTS) is a rare autosomal dominant inherited genodermatosis that is considered to be a phenotypic subtype of hereditary nonpolyposis colorectal cancer (HNPCC), commonly referred to as Lynch syndrome. We describe the clinical course of a 57-year-old female patient with MTS. She has a confirmed* HMSH2* mutation. Recently she presented with two nodular lesions. Histologic examination confirmed these lesions to be sebaceous neoplasms. The patient has a history of endometrial and colorectal adenocarcinoma as well as several nonspecific sebaceous lesions throughout her life. She has a confirmed extensive family history of MTS with both male and female family members harbouring either* HMLH1* or* HSMH2* mutations. Affected relatives have presented at different ages throughout their lives with cutaneous neoplasms and visceral malignancies, including malignancies rarely associated with MTS. MTS presents a diagnostic challenge for clinicians. The case demonstrates that the management of MTS, a potentially underreported syndrome, requires a multiprofessional approach incorporating vigilance, screening, and expert knowledge for successful diagnosis and potentially improved prognosis for patients and their families. The case also demonstrates the varied heritability of MTS and prompts the question of how MTS is expressed in succeeding generations.

## 1. Introduction

Muir-Torre syndrome (MTS) is a rare autosomal dominant inherited genodermatosis with a high degree of penetrance considered to be a phenotypic subtype of hereditary nonpolyposis colorectal cancer (HNPCC) or Lynch syndrome. MTS has been further described as a subtype of Lynch syndrome Type II. Lynch syndrome and MTS are derived from a heritable germline mutation in one or more DNA mismatch repair (MMR) genes, namely,* HMSH2 *and* HMLH1* [1–4]. Mutations of these human and mouse MMR genes are associated with microsatellite instability and neoplastic growth. In the context of MTS, mutations of these genes are associated with cutaneous sebaceous growths and systemic malignancies [5–8].

MTS is characterized by at least one cutaneous neoplasm and at least one visceral malignancy [1, 5, 6, 9–11]. Cutaneous neoplasms reported in MTS are of sebaceous etiology, nonspecific clinically, and rarely seen in the general population. Visceral malignancies commonly reported in MTS are of gastrointestinal and genitourinary etiology [3, 5, 7, 12]. MTS is a rare condition with only several hundred cases reported to date [13–15]. The majority of MTS cases are associated with mutations of* HMSH2* [5, 15]. We present a case that exemplifies the varied heritability of MTS (in terms of severity and age of onset) in succeeding generations as well as the importance of regular screening, vigilance, and the necessity of a multiprofessional approach for the effective detection and management of this rare and likely underreported condition.

## 2. Case

**Figure 1:**
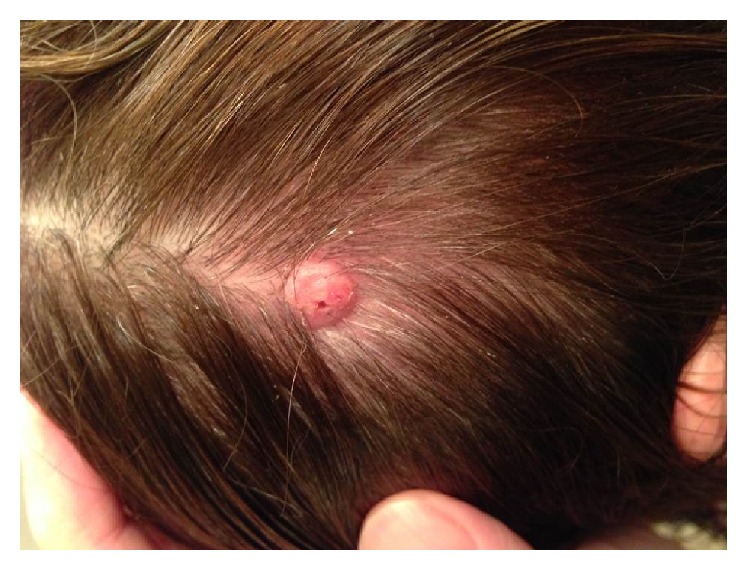
Nonspecific sebaceous lesion on the patient's scalp. Lesion was excoriated and removed prior to clinical evaluation.

A 57-year-old female with known Muir-Torre syndrome presented to their dermatologist with two nonspecific sebaceous lesions, each appearing as yellow erythematous nodules with cystic features. The two lesions on the back were removed: 0.5 × 0.5 cm mid back and 0.5 × 0.4 cm lower back. Histologic examination of the lesion on the patient's mid back revealed a sebaceoma and was described by the pathologist as a well circumscribed lobulated tumor with regional cyst formation. The cellular population consisted predominantly of basaloid cells with a variable sebaceous component. The second lesion obtained from the lower back was diagnosed as a sebaceous adenoma and was described as demonstrating papillary epidermal hyperplasia, sebaceous lobules with a basaloid rim, focal parakeratosis, and mixed inflammatory infiltrate. Immunohistochemistry was able to confirm an* HMSH2* mutation. Although being diagnosed with sebaceous neoplasms in the past, the patient has had several cutaneous lesions throughout her adult life that were not biopsied, including, most recently, an erythematous, nodular lesion on her scalp (Figure 1). This lesion had been excoriated and subsequently removed prior to clinical examination and therefore a specific diagnosis was not determined.

The patient had three previous internal growths. The patient's past medical history revealed that she was diagnosed with endometrial adenocarcinoma in 1993, for which she underwent a radical hysterectomy. She was 36 years of age at the time of the diagnosis. After this first malignancy, the patient, as well as many of her family members, underwent genetic testing. Several years later in 2008 she was diagnosed with intramucosal adenocarcinoma of the ascending colon, for which a right hemicolectomy was performed. The patient underwent regular colonoscopies and endoscopies every 6 months for several years following the discovery of this colonic adenocarcinoma. In 2014, during her most recent colonoscopy/endoscopy, a tubular adenoma was removed from the terminal ileum.

**Figure 2:**
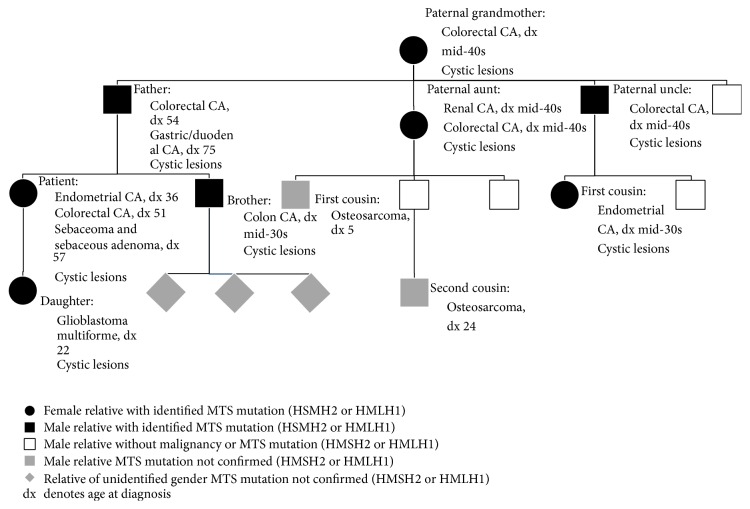
The patient's family history, including visceral malignancies and MMR mutation information.

The patient's family history documented several paternal relatives with sebaceous neoplasms and visceral malignancies (Figure 2). In 1989, her father was diagnosed with colon cancer at the age of 54 for which a resection of the affected portion of his large bowel was performed. Soon after her father's initial diagnosis in 1989, her paternal aunt was found to have rectal cancer and cancer of the kidney for which she was treated successfully by way of resection and radiation therapy. Her paternal uncle was diagnosed with colon cancer and treated successfully by way of hemicolectomy. Both her paternal aunt and uncle were in their mid-40s. Her father, paternal aunt, and paternal uncle had experienced sebaceous growths at different times throughout their lives. It was after the diagnosis of the visceral malignancies affecting the patient's father, aunt, and uncle, as well as the patient's first malignancy, that the patient's family (both first and second degree relatives over the age of 18, including the patient) underwent germline mutation analysis (serum). It was found that mutations of* HMLH1 *and* HSMH2* MMR genes, associated with MTS, were present in her family. Our patient was confirmed to have a mutation of the* HMSH2* gene. The patient's brother succumbed to colon cancer in his mid-30s and her daughter was diagnosed with Glioblastoma Multiforme (GBM) at 22 years of age. Both relatives had a confirmed* HMSH2* mutation and had experienced sebaceous growths throughout their lives. Interestingly, the patient's paternal aunt had three children, one of whom died of osteosarcoma at the age of 5. Further, one of her aunt's grandchildren was diagnosed with osteosarcoma at the age of 24 and treated successfully due to the early detection of the cancer. Neither family member submitted to genetic testing, nor could a propensity for osteosarcoma within the family be identified (Figure 2). The patient and her family undergo yearly dermatological examinations, colonoscopies, endoscopies, and other recommended screening.

## 3. Discussion

Muir-Torre syndrome is a rare autosomal dominant heritable condition with variable expression, characterized clinically by at least one sebaceous neoplasm and at least one visceral malignancy [5–7, 10, 11]. MTS has only been diagnosed in several hundred people worldwide, but it is maintained that many cases go unreported [13–15]. Lynch et al. [16] describe MTS as a subset of Lynch syndrome Type II. These authors consider MTS to be an extended pleiotropy of the genes involved in Lynch syndrome with increased and varied phenotypic expression, as evidenced by the visceral malignancies of varying etiologies affecting the patient and her family in the case presented [3, 16]. The mechanism of tissue specificity for neoplastic growth has yet to be identified [7]. In Lynch syndrome,* HMSH2 *mutations account for 40% of germline mutations associated with malignancy. In MTS, approximately 90% of mutations associated with malignancy involve mutations of the* HSMH2* gene [5, 17, 18].

Sebaceous neoplasms associated with MTS are nonspecific clinically. They usually appear on the face and other sebaceous gland regions [1, 6]. Sebaceous gland neoplasms commonly associated with MTS include sebaceous adenomas, sebaceomas, sebaceous carcinomas (malignant), and keratoacanthomas [5, 6, 19, 20]. It is recommended that patients presenting with these skin lesions have a detailed family history and undergo evaluation for visceral malignancies [1, 3, 6, 9]. In fact, MTS patients present almost ten years earlier with visceral malignancies than the general population [3].

The patient's daughter had Glioblastoma Multiforme, a rare MTS associated malignancy [7]. She was afflicted at a younger age relative to her mother by this aggressive grade IV malignancy. Similarly, the patient and her brother were afflicted by visceral malignancies earlier in life than preceding generations. From this, it would seem that succeeding generations are struck with arguably more aggressive malignancies earlier in life compared to MTS patients of preceding generations in this family. Of note, the patient's first and second cousins were also diagnosed with osteosarcoma relatively early in life (Figure 2). Although neither was tested for an MMR gene mutation, an association between* HMSH2* mutations and familial osteosarcoma has been made [21]. Generally, further research could help to establish prognostic data and provide further grounds for more vigilant and earlier screening in MTS families.

Consistent with the case described, a variable temporal relationship has been shown to exist between cutaneous sebaceous growths and visceral malignancies in MTS [15, 22, 23]. This, coupled with the nonspecific nature of the sebaceous neoplasms demonstrated by the case, can present a diagnostic challenge for clinicians. For this reason, patients and practitioners must be vigilant to detect internal malignancies at an early stage. A multiprofessional approach can help to diagnose, screen, and optimally treat these patients.
